# Highly-multiplexed SNP genotyping for genetic mapping and germplasm diversity studies in pea

**DOI:** 10.1186/1471-2164-11-468

**Published:** 2010-08-11

**Authors:** Chrystel Deulvot, Hélène Charrel, Amandine Marty, Françoise Jacquin, Cécile Donnadieu, Isabelle Lejeune-Hénaut, Judith Burstin, Grégoire Aubert

**Affiliations:** 1 INRA UMRLEG, BP 86510, 21065 Dijon, France; 2 GENOTOUL Platform, INRA chemin de Borde-Rouge BP52627 31326 Auzeville, France; 3 INRA SGV, UMR SADV, Estrées-Mons BP 50136, 80203 Péronne, France; 4 Euralis semences, Domaine de Sandreau, 31700 Mondonville, France

## Abstract

**Background:**

Single Nucleotide Polymorphisms (SNPs) can be used as genetic markers for applications such as genetic diversity studies or genetic mapping. New technologies now allow genotyping hundreds to thousands of SNPs in a single reaction.

In order to evaluate the potential of these technologies in pea, we selected a custom 384-SNP set using SNPs discovered in *Pisum *through the resequencing of gene fragments in different genotypes and by compiling genomic sequence data present in databases. We then designed an Illumina GoldenGate assay to genotype both a *Pisum *germplasm collection and a genetic mapping population with the SNP set.

**Results:**

We obtained clear allelic data for more than 92% of the SNPs (356 out of 384). Interestingly, the technique was successful for all the genotypes present in the germplasm collection, including those from species or subspecies different from the *P. sativum ssp sativum *used to generate sequences. By genotyping the mapping population with the SNP set, we obtained a genetic map and map positions for 37 new gene markers.

**Conclusion:**

Our results show that the Illumina GoldenGate assay can be used successfully for high-throughput SNP genotyping of diverse germplasm in pea. This genotyping approach will simplify genotyping procedures for association mapping or diversity studies purposes and open new perspectives in legume genomics.

## Background

Pea (*P. sativum*), an important cool-season legume crop, is both a source of dietary protein for animal feed and human food and a beneficial crop in cropping systems [1,2]. For these reasons, pea is destined to play a central role in sustainable agriculture. The development of this crop requires higher and more stably yielding varieties. The tools for molecular breeding in pea are currently scarce, despite its adoption as a model species for genetics since Mendel's era [3-5]. A broad range of DNA markers has been developed in *Pisum *including microsatellite [6,7], retrotransposon-based [8], and gene-anchored markers [9-12]. These markers have been used for diverse purposes: to build consensus genetic maps [7,11,13], survey genetic diversity [14-17], and detect Quantitative Trait Loci (QTLs) [18-20]. Each of these types of marker presents advantages and drawbacks. Retrotransposon-based markers reveal numerous loci at a time but are dominant. Gene-based markers used until recently low-throughput technologies genotyping one single locus at a time, but they do allow assessment of synteny with other legume species [21,22,10,11]. Microsatellite markers have been the most widely used in the recent years, due to their large number of alleles per locus and their facile use by single PCR. However, genotyping of large populations using this technique is still expensive and time consuming.

Different genotyping technologies have recently been developed to take advantage of the wealth of Single Nucleotide Polymorphisms (SNP) present in all eukaryotic genomes. In humans, SNPs make up about 90% of all human genetic variation and occur every 100 to 300 bases along the 3-billion-base human genome [23]. Similar studies in chicken showed a mean diversity of about 1 SNP every 200 bases for almost every possible comparison between 2 lines [24]. In plants, SNP are also very frequent, although their frequency seems to vary from one species to another. Zhu et al. [25] reported a frequency of nucleotide change of one SNP every 270 base pair (bp) on average in soybean while this frequency was found to be higher in maize (1 polymorphism every 60 bp) [26]. In *Pisum*, Jing et al. [27] reported one SNP every 20 bases in intronic regions using a set of 52 accessions representing the wild diversity of the genus. SNP markers, even though mostly bi-allelic, can be easily used for genetic and association mapping, to structure genetic diversity [28] or for genome-wide selection [29].

Many different techniques can be performed to genotype SNP markers, from the low-throughput allele-specific PCR [30] to high-throughput methods genotyping hundreds of thousands of SNP in parallel. Depending on the number of samples and markers of the project to be analysed, medium to high-throughput array-based SNP genotyping systems are now available, such as Illumina GoldenGate and Infinium, SNPStream from Beckman Coulter, MegAllele or GeneChip from Affymetrix (for a review, see [31]). The Illumina GoldenGate assay allows genotyping large collections of samples for a large number of SNP (96, 384, 768 or 1536 SNPs per assay) over a 3-day period with a high level of multiplexing [32]. Using two allele-specific primers located on the SNP and differentially labelled with Cy3 and Cy5, and a locus-specific primer recognizing both alleles addressed to a micro-bead and identifying the locus through a barcode, the technology allows multiplexed discrimination of the two alleles of any SNP locus in a single reaction.

In the last decade, high-throughput SNP genotyping has been extensively applied to human [33,23] or animal panels [24,34,35]. Some studies have also applied high-throughput SNP genotyping technologies to plants, mainly cereals [36-39], but also spruce [40] and legumes like soybean [41] or cowpea [42]. Most of these studies used the Illumina Goldengate assay. To date only a few have analysed plant germplasm collections using this technique. For cereals, Rostoks et al [36] characterised 102 barley genotypes representing mainly the West European cultivated diversity, and Akhunov et al. [37] genotyped 91 wild or cultivated lines of wheat with 96 SNP. For legumes, a collection of 96 soybean landraces was successfully genotyped with a set of 384 SNP [41]. These technologies always require a preliminary step of SNP discovery. The SNP detection methods are generally based either on (i) the discovery of electronic SNP in EST or shotgun genomic libraries [43] involving large sequencing programs, or (ii) the re-sequencing of PCR amplicons in different genotypes [25,40].

Little genomic sequence data is available for Pea (3900 genomic sequences in GenBank), and the number of available EST is also limited (18252 in Genbank, 9377 in the Crop-EST Database (IPK Gatersleben), if compared to the 2 millions and 1.5 million ESTs present respectively in the corn or soybean databases. Moreover, sequences are rarely present for different genotypes. Consequently, a very limited number of SNPs have been so far identified. In this paper, we compiled data obtained from the re-sequencing of gene fragments for different pea genotypes and from information present in different databases to identify SNPs and to build a 384 SNP marker set. We used the Illumina GoldenGate [32] and the Veracode technologies on a BeadXpress Platform [44], and genotyped a mapping population as well as a germplasm collection. This allowed us to test the suitability of this technique for a non-sequenced species, and to assess the efficiency of the defined SNP set for mapping or diversity studies in a large germplasm collection including accessions from different *Pisum *species and subspecies.

## Methods

### Plant material

Two different sets of plants were used for genotyping. The first consisted of one Recombinant Inbred Line (RIL) mapping population of 91 F6 plants (Pop9), developed by Single Seed Descent from the cross between the genotype 'China' (JI1491) and the cultivar 'Cameor'. The second was a panel of 373 *Pisum *accessions from different geographical origins, including modern cultivars, landraces and plants from wild populations, representing both cultivated and wild germplasm diversity (Additional file 1). This set also included parental genotypes of published mapping populations, namely JI281, JI399 and JI15 [45], cv 'Terese', K586 which is a mutant obtained from Torsdag [46,7] Champagne [7,20], JI296 and DP [12]. DNA was extracted from leaf tissue using a CTAB method as described by Rogers and Bendich [47]. The DNA concentrations were evaluated using the Quant-iT dsDNA BR kit (Invitrogen) measuring the Pico green fluorescence on an ABI7900 apparatus (Applied Biosystems). DNA concentrations were adjusted to 50 ng/μL for each sample.

### SNP discovery and selection

#### Two different strategies were used to identify SNPs

(i) Firstly, genomic, EST or cDNA pea gene sequences were selected from Genbank or the IPK Crop EST database http://pgrc.ipk-gatersleben.de/cr-est/index.php. Primers were then designed on these sequences in order to amplify and directly sequence genomic fragments in 2 to 12 pea genotypes, as described in Aubert et al. [11]. The sequences obtained were aligned using ClustalW, and potential polymorphisms were checked on the chromatograms.

(ii) Secondly, we searched for pea genomic sequences present in Genbank for different genotypes. Such data has been produced for cross-species legume comparative mapping [48], gene diversity studies [27] or studying a specific gene [49-53]. Sequences were retrieved and aligned with ClustalW in order to visualize SNP.

A preliminary list of 520 SNPs was selected using the BeadXpress primer design (Illumina, San Diego, CA) using as criterion the absence of any other SNP in the 30 bp segment flanking the SNP analysed and in the 30 bp zone located 20 bp downstream of the SNP. A designability rank score (0 to 1) was calculated for each SNP by Illumina. 384 SNP with designability scores between 0.401 and 0.999 were finally selected which maximised both the number of genes represented in the set and the diversity when more than one SNP was selected for a gene. Three primers were then designed by Illumina for each SNP locus, using the Veracode Assay Designer software. Sequence and primer information for the 384 SNPs are listed in Additional file 2.

### SNP genotyping

The GoldenGate assay is based on the use of 2 allele-specific and one locus-specific oligonucleotides per SNP locus. After hybridisation of these oligonucleotides on the template DNA, an allele-specific extension/ligation step is performed and is followed by a PCR reaction with three universal primers.

PCR products are labelled with Cy3 or Cy5 depending on the allele, and contain an Illumicode address sequence specific of the locus. Each address sequence corresponds to a glass Veracode micro-bead, which bears a locus-specific barcode. Thereby, every SNP locus is identified by its IllumiCode address and alleles at the SNP locus are discriminated by their fluorescent signals. The Illumina OligoPool Assay was performed according to the manufacturer's protocol, as described by Fan et al. [41]. 250 ng of genomic DNA was used for each genotype, with control DNA of genotypes 'Cameor' and 'China' on each plate. After amplification, the PCR products were hybridized to the Veracode beads via the address sequence for detection on a Veracode BeadXpress Reader [44]. For each SNP, the amplification product for homozygous genotypes displays normally a signal in either the Cy3 or Cy5 channels, whereas the heterozygous genotype at this locus should display a signal in both channels. The automatic allele calling was done using the Illumina Genecall software with a GeneCall threshold of 0.25. The software assigns three clusters on a graph based on the fluorescence obtained. Different indexes were calculated by the software. Several were used to check the automated genotype calling and the sample clustering: (i) the Call Rate is the number of SNP successfully genotyped for each sample; (ii) the GenTrain Score evaluates the confidence of the genotyping for one SNP on all samples. It depends on the distance between the 3 clusters and the fluorescence intensity; (iii) the Gene Call Score (GC Score) is a confidence score of the genotyping of each point. It depends on the intensity of fluorescence and the distance of the point from the centre of the cluster on the graph. The homozygous and heterozygous clusters were checked visually and revised, and only the most reliable calls were retained. A quality mark was then given to each SNP as follows: (0) Failed; (1) No polymorphism detected; (2) Polymorphism detected but low fluorescence or weak cluster separation and (3) Clear genotyping and good cluster separation but some accessions (> 10%) were not genotyped or formed a cluster corresponding to a third allele, and (4) Excellent genotyping. The consistency between the SNP genotyping obtained using the GoldenGate assay and the Sanger sequencing was checked for each SNP on the genotypes for which the sequence was available. This allowed assessment of GoldenGate genotyping accuracy.

### Genetic mapping

Using 35 framework markers from Aubert et al. (2006) distributed over all linkage groups, the markers were placed using the try, place and ripple command of MAPMAKER/EXP version 3.0b [54]. Default LOD and distance threshold were used. The Haldane function was used to calculate centiMorgan (cM) distances. The map was drawn using MapChart [55].

## Results

### Design of the pea Illumina Veracode 384 SNP set

Genomic sequence information was obtained in our labs for 334 different genes using 2 to 12 different genotypes. In parallel, we retrieved genomic sequence data available for at least 2 genotypes from Genbank for each gene. Combining these two sources of information allowed us to identify 2850 SNP (1170 from Genbank alignment) in 308 genes.

We selected 520 of these SNPs that matched the Illumina criterion of absence of other known SNPs in their vicinity and with sufficient sequence information upstream and downstream of the SNP. Of these, 142 came from the information retrieved from Genbank, and 378 were new. A designability score was given to each SNP by Illumina, with the score ranging from 0 to 1.0, where a score < 0.4 predicted a low success rate, between 0.4 and 0.6 a moderate success rate, and > 0.6 a high success rate for the conversion of a SNP into a successful GoldenGate assay. Out of the 520 SNP, 363 had a score > 0.6 (designability rank = 1), and 48 ranked between 0.4 and 0.6 (designability rank = 0.5). The pea Illumina GoldenGate assay finally consisted of 346 SNP with a designability rank of 1 and 38 SNP with a rank of 0.5 (mean designability score of 0.821). The 384 SNP markers represent 205 different genes involved in various physiological processes such as cold acclimation, nitrogen and carbon metabolism or symbiosis (Additional file 2). Map positions were known for 110 of these genes, with 15, 17, 24, 14, 15, 13 and 12 genes respectively placed on linkage groups 1 to 7 (See Additional file 2).

### Polymorphism and allele call for the different SNP

For all SNPs, genotyping was checked visually, using Sanger control sequences and taking advantage of the defined allelic structure of the RIL mapping population. The SNPs were graded 0-4 according to the quality of the polymorphism detected and to the quality of the genotyping and allele detection (Additional file 3). The vast majority of SNPs (325 out of 384) gave a clear genotyping (quality mark of 3 or 4). Of these, 301 were successful for nearly all accessions (> 90% of the collection, quality mark of 4). Thirty one SNP had either an ambiguous cluster separation or a low GenTrain score (Quality mark of 2). Thirteen did not show any polymorphism (Quality mark of 1). Three hypotheses can be invoked to explain the absence of detected polymorphism: (i) false SNPs, resulting from possible sequencing mistakes (ii) rare SNPs, not present in our collection of accessions (this case is possible if the sequences retrieved to define the SNP were obtained from genotypes not present in our panel), or (iii) the incapacity of the technique to discriminate a SNP at this locus. The absence of cluster separation can be due, for example, to a non allele-specific match of the primers. Re-sequencing of these thirteen loci would be needed to distinguish between these hypotheses. Fifteen SNPs could not be genotyped (Quality mark of 0). Out of these 15 SNP, 7 had a SNP score between 0.4 and 0.6, while the score of the 8 remaining was over 0.6.

**Figure 1:**
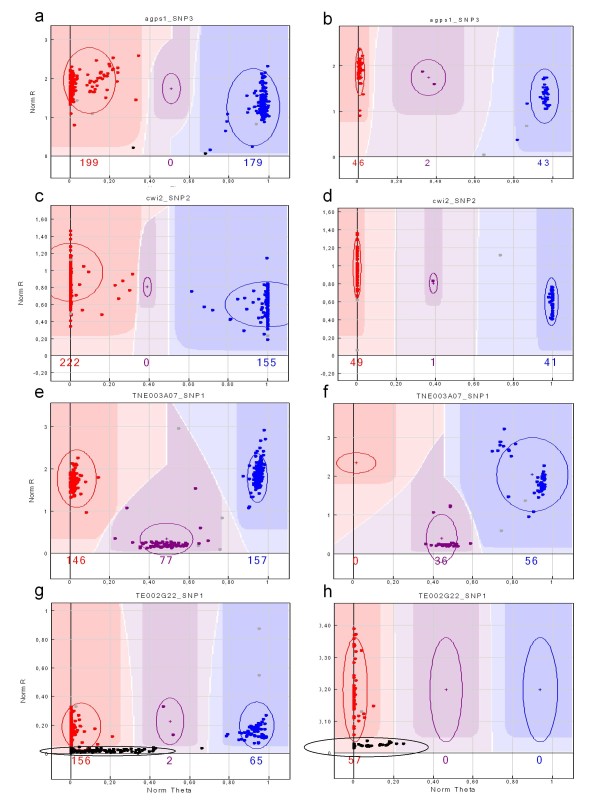
**Example of graphical display obtained with the Illumina Gene Call Software for 4 different SNPs:** (a, c, e, g) germplasm collection genotyping, (b, d, f, h) RIL population genotyping. The 4 SNPs are (a,b) agps1_SNP3, (c,d) cwi2_SNP2, (e,f) TNE003A07_SNP1, (g,h) TE002G22_SNP1. The data points colour codes for the call (red = AA, purple = AB, blue = BB). Genotypes are called for each sample (dots) by their signal intensity (Norm R, y-axis) and Allele Frequency (Norm Theta, x-axis) relative to canonical cluster positions (dark shading) for a given SNP marker. For SNP TNE003A07_SNP1 (e,f), the purple points do not correspond to heterozygous plants but to a third allele. For SNP TE002G22_SNP1 (g,h), the black dots correspond to an additional null allele.

In the germplasm collection, most of the SNP yielded two clear main clusters representing the two homozygous genotypes, with sometimes a small additional cluster in the middle of the graph corresponding to heterozygous genotypes. This was expected for this type of population, mainly constituted of homozygous lines. Two examples of such a cluster separation are given in Figures 1a and 1c. When the SNP was polymorphic between parental genotypes 'China' and 'Cameor' and was therefore segregating in the RIL population, we observed a similar profile for the RIL population (Figures 1b and 1d) as for the germplasm collection. In these cases, we were able to compare the cluster separation in the RIL population and genetic resources collection: a larger intra-cluster variability was often observed when genotyping the collection of 373 accessions (Figures 1a and 1c) as compared to the clusters observed for the same alleles in the RIL population (Figure 1b and 1d), probably due to additional polymorphism near the SNP in the genetic resources collection. Interestingly, in some rare cases, 3 main groups of alleles were detected, the third cluster being positioned between the two first ones at the bottom of the graph. An example of such a cluster separation is given in Figure 1e with TNE003A7_SNP1. The segregation of the third allele of the same SNP was observed in the RIL population (Figure 1f). The sequencing of the corresponding gene fragment in genotypes belonging to the middle cluster showed the presence of a 14 bp deletion surrounding the SNP locus. This explained the reduced signal obtained for the genotypes with this deletion. In a few other cases (indicated in Additional file 3), a third null allele was detected in addition to the ones corresponding to the two main homozygous clusters, as for TE002G22_SNP1 (Figure 1g). In that case, the cluster corresponding to the null allele was closer to the cluster of genotypes having a "G" base genotype at this SNP locus. As this null allele segregated in the RIL population (Figure 1h, Null allele for genotype 'China'), we could observe a perfect co-segregation of this marker with another polymorphic SNP of the same gene (TE002G22_SNP3). Sequencing the gene fragment in some accessions exhibiting the null allele showed that they harboured not only the G base at the SNP locus but also a mutation downstream of the SNP at the penultimate base of the locus-specific primer. The lower fluorescence detected for the genotypes having the mutation is presumably due to the resulting primer mismatch.

### Genetic mapping of the SNP in the 'China' X 'Cameor' RIL population

**Figure 2:**
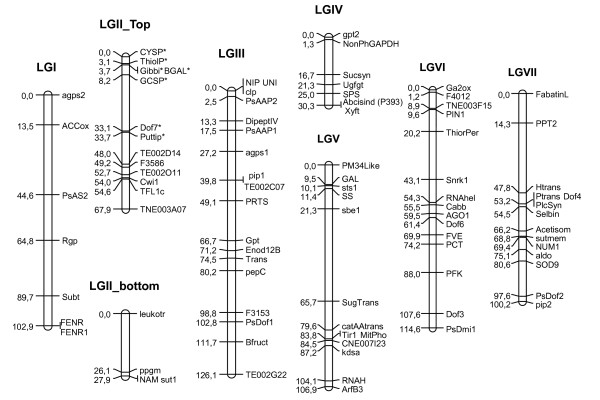
**Genetic map of 'Cameor' X China RIL population (Pop9)**. Haldane distances in centimorgans are indicated on the left of linkage groups and locus names on the right. Markers showing significant segregation distortion (P < 0.01) are indicated by an asterisk.

**Figure 3:**
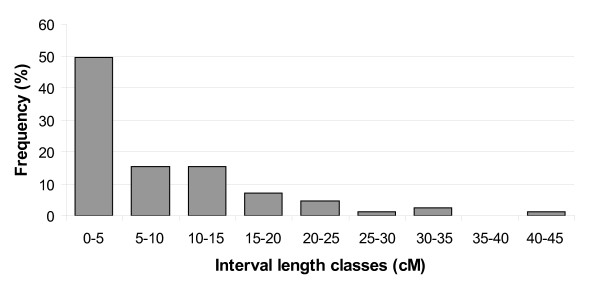
**Interval length distribution (in centimorgans) between the 91 markers of the Pop9 map**.

In order to investigate the applicability of the SNP set for genetic mapping in pea, we genotyped 91 F6 RIL derived from the cross between 'China', a Chinese accession, and 'Cameor', a European garden pea cultivar. Out of the 384 SNPs from the Illumina Veracode set, 144 SNPs were polymorphic among these two genotypes, representing 95 gene sequences. When there was more than one polymorphic SNP per gene, the different SNPs gave similar genotyping results, which confirm the genotyping accuracy as no recombination events are expected in one gene sequence for a population of this size. Consequently, only one SNP per gene was used for further genetic mapping. The genetic map for Pop9 (Figure 2) comprised 91 loci organised into 8 linkage groups. Four markers (rbcs, agpl1, cwi2, TE002I24) remained unlinked. Seven markers on the top of LGII showed a significant segregation distortion (chi2, P < 0.01) in the RIL population. The average distance between markers is 8.2 cM, and 65% of the intervals between markers are smaller than 10 cM (See the interval length distribution on Figure 3). For 54 genes out of the 91, the map position in Pop9 confirmed previous mapping results (Additional file 2). For the 37 remaining genes, this is the first report of their position on the pea genetic map.

### Gene Call and allele frequency estimates in the germplasm collection

The germplasm collection consisted mainly of *P. sativum *ssp *sativum *accessions, complemented by accessions of ssp *elatius*, *abyssinicum *and two *P. fulvum *accessions. Interestingly, call rates (proportion of scorable SNPs for a given genotype among the 356 useable SNPs) were quite high (see Additional file 1) and very similar among the different subspecies, including between *P. sativum *and *P. fulvum*, indicating that SNP markers were successfully amplified and genotyped in diverse germplasm.

In order to provide some information about the usefulness of the 384 SNPs markers for genetic studies, allele frequencies were calculated for each SNP in the germplasm collection. As SNP with equilibrated allele frequencies are more likely to be polymorphic between two genotypes than SNPs with rare alleles, this can be a criterion for selecting SNPs for genetic mapping or diversity survey. The distribution of minor allele frequencies for the useable polymorphic SNPs was uniform between classes [0, 0.1[,[0.1, 0.2[,[0.2, 0.3[,[0.3, 0.4[,[0.4, 0.5] (data not shown),. Only 72 SNPs (20%) had minor alleles with frequencies lower than 0.1.

### Evaluation of the set to genotype existing mapping populations

In order to evaluate the potential of the SNP set for use in genotyping other mapping populations, the numbers of markers polymorphic between JI15 and JI399, JI281 and JI399 [45], Terese and K586, a mutant of Torsdag [46], JI296 and DP [12] and Champagne and Terese [7,20] were evaluated, as they are parental genotypes of published mapping populations and are included in our germplasm collection (Table 1). Between 110 and 148 SNPs (on average 36.1% of the 356 successful SNPs), representing 79 to 100 genes (on average 43.5% of the 200 genes), were polymorphic in each of the 5 populations. Depending on the population, between 39 and 53 markers were potential bridges with the Pop9 map.

**Table 1:** Potential polymorphism in different mapping populations

	**JI15 X JI399 **[45]	**JI281 X JI399 **[45]	**Terese X Torsdag **[46]	**Champagne X Terese **[7,20]	**JI296 X DP **[12]
Polymorphic SNPs	110	139	148	120	126
Genes potentially mapped	69	99	100	79	88
Bridge gene markers with pop9 map	39	53	52	48	51

Number of polymorphic markers and genes from the 384 SNP marker set in 5 published genetic mapping populations.

## Discussion

### The GoldenGate SNP assay is well-suited for genotyping a wide germplasm collection

In this paper, we have demonstrated the suitability of a 384-SNP GoldenGate assay, for genotyping both a genetic mapping population and a genetic resources collection of pea. Despite the wide diversity of the germplasm collection and the presence of *P. sativum *wild germplasm and of two *P. fulvum *accessions, most SNP markers were amplified and genotyped. Genotyping data were obtained for 356 out of the 384 SNP of the set, a success rate of 92.7%. 325 SNPs (84,7%) gave excellent genotyping results according to the criteria defined by Close et al.[39], which is comparable with the 89% and 90% success rates reported respectively in soybean [41] and in barley [36] using the same genotyping technology. The mean GenTrain score, which is a measure of the reliability of the SNP detection based on the intra-and inter-distribution of genotypic classes [32], was 0.63, and was never below 0.25 for any individual SNP (Additional file 2). We also compared these two parameters based on the preliminary designability rank given by Illumina. The conversion of an SNP into a successful GoldenGate assay is predicted to be unlikely, likely or very likely when the designability rank is 0, 0.5 or 1 respectively. Success rate and GenTrain scores were both higher for SNPs with a designability rank of 1 (respectively 94% and 0.64) than for the SNPs with a designability rank of 0.5 (81% and 0.57), demonstrating the relevance of this criterion for a preliminary selection of SNP.

The reliability of the technique was also evaluated by comparing the results of the GoldenGate SNP genotyping with Sanger sequencing data for a few genotypes. The results were consistent between the two techniques in all cases. This is also reinforced by the concordance of genotyping results for different SNPs of a same gene in the mapping population. Our study shows that the GoldenGate genotyping is very reliable, as also demonstrated in wheat [37]. We have also shown that the technique can reveal hitherto undetected genetic diversity, by distinguishing for some SNP a third allele in addition to the two previously identified alleles.

In addition, the screening of the SNP set on the germplasm collection gives valuable information for selecting for further use SNP markers with clear bi-allelic profiles as described by Close et al. [39].

### An efficient tool for integrating genetic maps

To test the suitability of the SNP set for mapping, we genotyped the recombinant inbred line Pop9 population. We were able to assign the linkage groups obtained by comparing them with the pea composite map previously published [11]. Two groups corresponded to LGII, while the 6 other groups corresponded to LGI, III, IV, V, VI, and VII. The Pop9 map covers about 680 cM, and represents approximately 70% of the pea map [11]. While LG1 and LG6 were totally covered, some chromosomal regions lacked polymorphic SNP markers in comparison to the map cited above, such as the middle of linkage group II, the top extremity of LGIII, and the bottom extremities of LGIV and LGV. Consequently, a few distal markers remained unlinked, for example agpl1_SNP2 (top of LGIII), cwi2_SNP2 and rbcs_SNP3, respectively, at the bottom of LGIV and LGV [11]. Marker order was conserved without exception for all linkage groups between the Pop9 map and the map of Aubert et al. [11]. In addition, 19 gene markers from other maps [10,12,13,22,27] were mapped in Pop9 to their expected linkage groups, providing anchor markers with these maps. Furthermore, information on the map position of 37 gene-anchored markers was obtained for the first time. Two of these markers, FENR (primers originally designed on a *Medicago truncatula *sequence[22] and FENR1 (primers designed on a *P. sativum *sequence, this study) mapped at exactly the same position. As both related sequences encode for a Ferredoxin-NADP reductase, this suggests that both markers correspond either to the same gene, or to two genes duplicated at the same locus. This demonstrates the utility of combining different maps to permit integration of a maximum of mapping data.

To investigate further the potential of the set as a source of new markers for genetic mapping we looked at the predicted number of markers it could provide in five existing populations. Our data showed that an average of 130 polymorphic SNPs, representing 87 genes, can be expected. Out of the 200 genes represented in the set, 143 were polymorphic in at least 2 of the 6 populations, showing the usefulness of the set in providing bridge markers and for comparing different pea maps. As the SNPs are linked to a gene sequence, they are also useful markers for studying synteny with other legume species [22,11,42].

Although the assay is a good tool for quickly providing a genetic map for a pea mapping population, the number of markers would have to be increased to obtain a saturated genetic map. This can be done either by increasing the number of SNPs in the set and/or by selecting only SNPs polymorphic between the parental lines in the panel. In our case, a set of 384 random SNP generated a map for Pop9 covering approximately 70% of the consensus map obtained in previous studies. Theoretically, 99% coverage should be obtained by using a higher multiplex custom assay of 1,536 SNP. However, more pea genome sequence information would be needed in order to increase the number of SNPs available and hence enlarge the size of the assay.

## Conclusion

Our results demonstrate the suitability of the GoldenGate assay for high-throughput SNP genotyping to characterise collections containing diverse germplasm or to rapidly establish genetic maps connected with pre-existing ones, and thus open new prospects for *Pisum *genomics. The use of next-generation sequencing technologies associated with techniques enabling to target specific regions [56] should allow sequencing of large regions of the pea genome followed by re-sequencing of these regions in different genotypes. This strategy should reveal thousands of SNPs that can be genotyped and mapped in different populations. The genotyping quality (Cluster separation [39], minor allele frequency) and map position data obtained for the different SNPs will help in the design of different panels utilisable for building consensus genetic maps, to study diversity, for positional cloning or in association mapping studies.

## Authors' contributions

CDe, AM, CDo genotyping and evaluation of the genotyping technology. HC germplasm collection and DNA preparation. FJ, ILH resequencing and SNP discovery. GA design of the SNP array. CDe genotyping data analyses. JB genetic mapping and funding. JB, GA project design, manuscript preparation and overall supervision. All authors have read and approved the final manuscript.

## Supplementary Material

Additional file 1 **Table S1-List of Pisum accessions used in this study**. Recalculated call rates correspond to the proportion of successful SNP genotyped for each genotype. Click here for file

Additional file 2 **Table S2-Information on the 384 SNP Illumina GoldenGate marker set**. This includes related accession number, sequence surrounding the SNP, preliminary designability score and rank, and primers used in the assay [57,58]. Click here for file

Additional file 3 **Table S3:Genotyping scores obtained with the Illumina GoldenGate assay for the different SNP**. Click here for file
